# A Systematic Review on the Prevention and Control of Opportunistic Infections in Patients With Chronic Lymphocytic Leukemia Complicated by Richter’s Transformation

**DOI:** 10.7759/cureus.22927

**Published:** 2022-03-07

**Authors:** Hadia Arzoun, Mirra Srinivasan, Stephanie Sandoval, Bridget Lee

**Affiliations:** 1 Internal Medicine, St. Bernard's Medical Center, Jonesboro, USA; 2 Internal Medicine, California Institute of Behavioral Neurosciences & Psychology, Fairfield, USA

**Keywords:** chronic lymphocytic leukemia (cll), immunosuppression, prophylaxis management, prevention and control, opportunistic infections

## Abstract

Chronic lymphocytic leukemia (CLL) is one of the most commonly occurring types of leukemia among the elderly population, contributing to an increased vulnerability to infections that are especially prolific in the immunosuppressed and the risk of rapid progression of the disease into a more aggressive manifestation of large cell lymphoma, a process called Richter’s Transformation (RT). CLL alone predisposes patients to develop infections; however, the additional complication of RT decreases survival and makes the prevention and control of infection for the CLL patient even more challenging. However, research that exists on preventing and controlling infection in CLL patients with RT is relatively limited. In most cases, studies have focused on the prevention of infection in CLL patients in general and with no reference to the progression of RT.

Considering the dearth of research on infection prevention and control for patients with CLL complicated by RT specifically, the following review examines existing research in addressing the prevention and control of infection in CLL patients with RT and patients in general. The authors explored multiple databases such as PubMed, Google Scholar, and Science Direct. The ultimate focus of this study was to lay a fundamental understanding in preventing and controlling infection in CLL patients. After analyzing several studies, it can be concluded that identifying infections, even if rare, is a crucial aspect of managing CLL patients. A broad range of differential diagnoses should be sought in cases presenting with refractory CLL as well and management of infections before, during, or after CLL treatment should be considered.

## Introduction and background

By its simplest definition, chronic lymphocytic leukemia (CLL) manifests in the “clonal proliferation and accumulation of mature and typically CD5-positive B-cells in the blood, bone marrow, lymph nodes, and spleen” [1]. CLL accounts for around a quarter of all new leukemia cases and affects roughly one in 175 people at some point in their lives [2]. Infection prevention and control is especially critical in CLL patients because it manifests predominantly in elderly adults, increasing their vulnerability to opportunistic infections and complications leading to death. Richter’s Transformation (RT) is a secondary complication of CLL, which develops in as much as 5% to 10% of CLL patients and is indicated by histology most commonly marked by the presence of large B-cell lymphoma, in some cases, by the development of Hodgkin’s lymphoma, and in even fewer cases, by B-prolymphocytic leukemia [3].

The prevention and control of opportunistic infections like those that occur in patients with a compromised immune system are developed and implemented in order to improve immunity and reduce the long-term morbidity that treatment and RT contribute to. Considering that infection prevention and control protocols are imperative for the CLL patient with or without a “large volume of readily available primary tumor cells,” it is reasonable to assume that those protocols would be similarly applied in CLL patients with RT, even those who present with RT on their initial pursuit of medical care and screening [4]. It is important to note that immediate treatment of CLL with chemo or other cytotoxic therapies is not always indicated, which is significant because the research will show that therapies, in general, contribute to infections in this population.

Treatment, however, is critical if and when the CLL patient progresses to RT [5]. It should be noted as well that, once a therapeutic option is initiated, for example, ibrutinib, even a temporary pause in its course can work to instigate the “release of inhibition of B-cell receptor signaling” and induce RT [5]. Nevertheless, CLL patients are in an immunosuppressed state regardless of stage and incidence of RT, with any difference in how the prevention and control of infection are meted out in its progression being dependent on the initiation of cytotoxic therapies and the incidence of RT, which diminishes immune-competence even further [6]. An investigation of how those differences might look in terms of infection prevention and protocols follows.

Methodology

**Figure 1:**
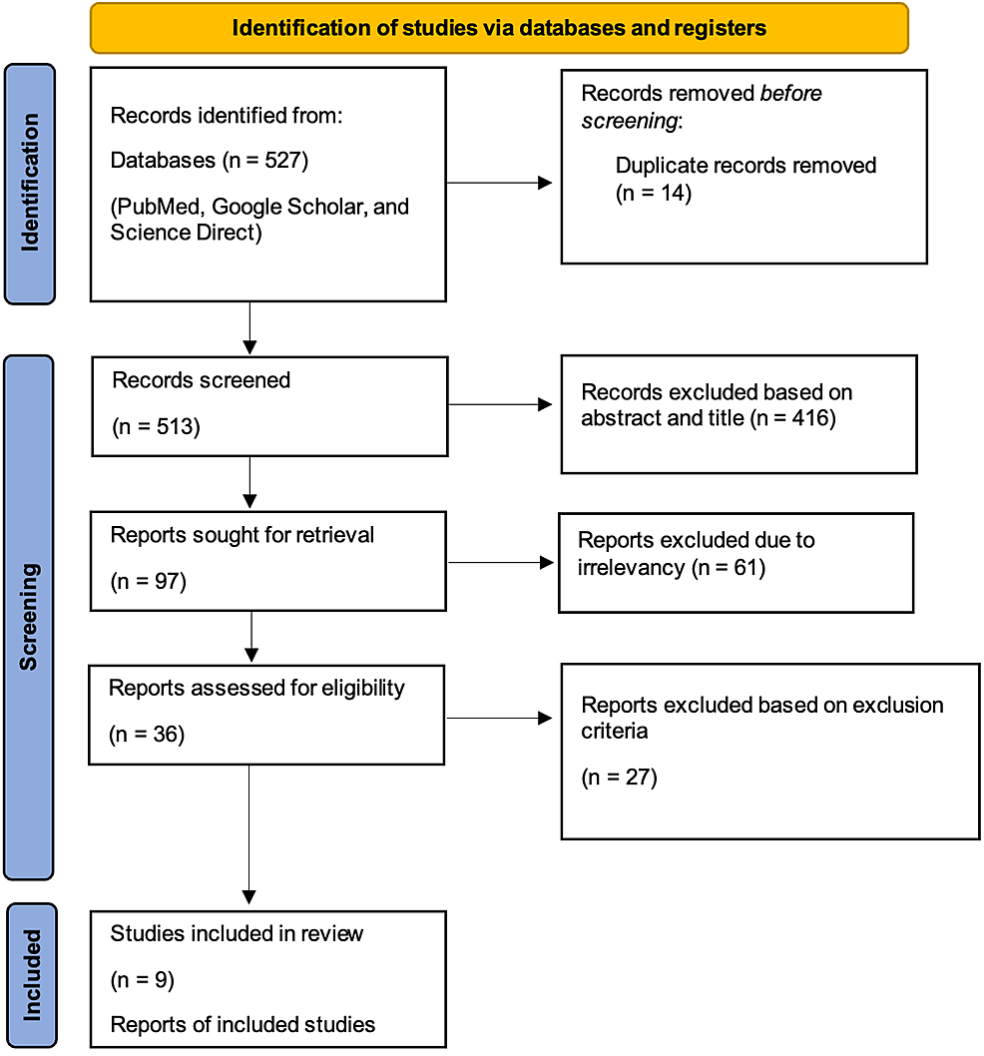
PRISMA Flowchart PRISMA: Preferred Reporting Items for Systematic Reviews and Meta-Analysis

This review article follows the Preferred Reporting Items for Systematic Reviews and Meta-Analysis (PRISMA) guidelines and principles 2020 [7]. A literature search with appropriate keywords, which are listed below was done in PubMed, Google Scholar, and Science Direct, and the search yielded 527 articles in total. After removing 14 duplicates, 513 records were screened, 416 reports were omitted based on the abstracts and titles, and 97 articles were sought for retrieval, among which 61 reports were omitted due to irrelevant study designs and methods. Twenty-seven reports were omitted based on the inclusion and exclusion criteria, and a final of nine reports were included as eligible studies after satisfying the respective quality assessment. This process was carried out by two authors independent of each other, and in cases of discrepancies among the authors, a third author was approached in order to find common ground. The search process is depicted in Figure 1 in the form of a PRISMA flowchart [7].

Keywords

This systematic review employed regular and medical subject heading (MeSH) keywords using the Boolean scheme.

Regular Keywords were Opportunistic infections; prevention and control; prophylaxis management; chronic lymphocytic leukemia/CLL; immunosuppression.

MeSH Keywords were Infection control OR ((“Opportunistic Infections/analysis” OR “Opportunistic Infections/complications” OR “Opportunistic Infections/immunology” OR “Opportunistic Infections/prevention and control” OR “Opportunistic Infections/therapy”)) AND Chronic lymphocytic leukemia OR (“Leukemia, Lymphocytic, Chronic, B-Cell/complications” OR “Leukemia, Lymphocytic, Chronic, B-Cell/immunology” OR “Leukemia, Lymphocytic, Chronic, B-Cell/metabolism” OR “Leukemia, Lymphocytic, Chronic, B-Cell/physiopathology” OR “Leukemia, Lymphocytic, Chronic, B-Cell/therapy”).

Inclusion and exclusion criteria

The inclusion criteria for the review article consist of full-text reports in the English language from the past 10 years including review articles and case reports as the study designs. Non-full-text, non-English language, non-human studies, reports prior to 2012, and reports with all other study designs were eliminated.

Results

Table 1 enumerates the individual characteristics of the final included reports for this review article [5,8-15].

**Table 1:** Summary of the Final Included Studies SANRA: scale for the quality assessment of narrative review articles; CLL: chronic lymphocytic leukemia; JBI: Joanna Briggs Institute; SMKIs: small molecule kinase inhibitors

Author	Year	Study Design	Quality Appraisal Tool	Scores Awarded	Conclusion
Whitake et al. [8]	2014	Review	SANRA checklist	>9; include	It is believed that novel vaccinations, alternate immunization schedules, and the use of vaccine adjuvants could improve vaccine responses in CLL patients and minimize infection-related fatalities in this immunocompromised group.
Lachance et al. [9]	2016	Review	SANRA checklist	>9; include	In individuals with hypogammaglobinemia, a history of infection, or both, immunoglobulin replacement therapy has been reported to prevent bacterial infections and related hospitalizations.
Morrison et al. [10]	2016	Case report	JBI checklist for case report	>7; include	Although acanthamoebiasis-related cutaneous lesions are extremely rare, they should be included in the differential diagnosis of necrotic cutaneous lesions in immunocompromised individuals. When started empirically, multiagent therapy regimens have been more effective than single-agent regimens; however, infections of the central nervous system are virtually always fatal.
Tadmor et al. [11]	2018	Review	SANRA checklist	>9; include	In CLL patients, proactive and reactive infection management is a major emphasis of treatment. Infections associated with conventional chemotherapy, monoclonal antibody immunochemotherapy, target treatments using B-cell receptor pathway inhibitors, and Bcl-2 antagonists must be addressed and treated promptly.
Katragkou et al. [12]	2018	Review	SANRA checklist	>9; include	A considerable amount of research suggests that therapies supporting or stimulating immune response might be viable options for immunocompromised individuals. Immunoglobulin treatment and prevention of infections in immunocompromised patients have shown beneficial results.
Teh et al. [13]	2018	Review	SANRA checklist	>9; include	Ibrutinib treatment, whether used alone or in combination with other chemo-immunotherapies, has not been linked to an increased risk of infection. Idelalisib, on the other hand, is linked to a two-fold increased risk of severe infection and opportunistic infections. Venetoclax does not appear to be linked to an increased risk of infection. Prophylaxis, monitoring, and vaccination are essential aspects of infection prevention.
Stein et al. [14]	2018	Case report	JBI checklist for case report	>7; include	Before initiating ibrutinib or other SMKIs, clinicians should be aware of medical comorbidities that may predispose to opportunistic fungal infections and look into preventative options.
Deerwester et al. [15]	2020	Case report and review	JBI checklist for case report	>7; include	Cytomegalovirus infection should be considered in the differential diagnosis of pustulonodular skin lesions, and early identification is crucial due to the danger of death, increased risk of mortality especially in immunocompromised individuals with CLL.
Baral et al. [5]	2021	Case report and review	JBI checklist for case report	>7; include	Physicians should consider using filgrastim as a growth factor for neutropenia and be on the lookout for early symptoms of infection to ensure appropriate antibiotic coverage. Infection of concern: Candida and Aspergillus

## Review

The following section focuses on the prevention and control of infection in CLL patients, including the immunodeficiency aspect, the vaccines that have attempted to alleviate these infections, and finally, the impact of Richter transformation is highlighted as well. The limitations of this study are also discussed at the end of this section.

Prevention and control of infection

Infections and their complications are the primary cause of morbidity and death in CLL patients at a rate of as much as 80% morbidity among this population and contributing to a mortality rate of 60% throughout the disease process [4]. The use of cytotoxic therapies like chemotherapy further undermines an immune system that is already weakened by CLL in the patient, making the risk of infections and their complications even more likely and more aggressive, and making infection prevention and control even more critical than ever [4]. Infections complicating CLL alone or with RT can occur because of three different reasons, the outcomes of which can be prevented and controlled with appropriate protocols. These include infections that result from a diminished immune response (immunodeficiency) that follows the development of CLL (and RT malignancy if present), infections that result from immunosuppression following treatment regimens for CLL (and RT malignancy if present), and/or infections that result from direct exposure to or contact with infectious pathogens.

In many cases, the protocols identified for preventing and controlling infections in CLL and CLL w/RT patients are the same or similar across these three facilitators. The immunodeficiencies that identify the first two entries above are similar by the outcome of immunosuppression in CLL/CLL-RT patients, however, the first reason is inherent in or attributed to the disease while the second is related to the therapies that are used to treat CLL/CLL-RT [11]. The third reason speaks to the fact that CLL/CLL-RT patients, like other immunosuppressed individuals, are vulnerable to opportunistic infections through exposure to and/or contact with sources of infection, however, to a greater degree because of advanced age and comorbidities that often come with age including diabetes, kidney, and circulatory problems, and other issues that increase the likelihood of infections and their complications [11].

Preventing infection attributed to immunodeficiency

CLL patients are regularly at risk for infection and related complications by the immunodeficiency that is inherent in the disease itself. Immunosuppression relative to the development of CLL can be attributed to different causative factors, one of the most prominent of which is hypogammaglobulinemia, which is a marker of the immune system’s inability to produce immunoglobulin, the antibodies that are vital to fighting both viral and bacterial infections [5,11]. Infections that impact patients with “early, untreated CLL” are those that are related to hypogammaglobulinemia (HGG) and generally confined to “infections by encapsulated bacteria” [16]. Immunoglobulin replacement therapy (IGRT) is often submitted as a mechanism for the prevention and control of infection in CLL patients, a proposition that can be fairly generalized to CLL patients when considering that patients with non-Hodgkin’s and Hodgkin’s lymphomas are regularly treated with IGRT.

The subject of HGG, immunosuppression in malignancies like CLL, and the proposal that IGRT can work to prevent and control infections and their complications, however, are regularly debated. This is in spite of the fact that HGG gets progressively worse proportionate to the worsening of the disease over time and the replacement of immunoglobulin can go a long way in mitigating the risk of infection in CLL patients [9]. There is compelling evidence for the efficacy of IGRT as a mechanism for preventing opportunistic bacterial infections in CLL patients. One study speaks has shown recent support for this protocol, not the least of which are the results of studies demonstrating that immunoglobin administered intravenously or subcutaneously is associated with fewer bacterial infections in patients with CLL [9]. One of the shortcomings of IGRT is that it shows little if any efficacy in preventing or controlling viral infections, however, it can still support the prevention or control of bacterial infections that are secondary to viral infections, i.e., infections that work to complicate viral pneumonia. What cannot be ignored about the notion of IGRT being instrumental to the prevention of infections in CLL/CLL-RT patients is the fact that such infections are largely bacterial in nature and, unlike viral infections, are the ideal impetus for the initiation of Ig intravenous transfusion for their prevention and control [9].

Preventing infection subsequent to CLL, CLL-RT treatment

The perpetuation of established therapies as well as the growth of new treatments has presented significant improvements for patients with CLL/CLL-RT, especially in the management of the disease. At the same time, however, they have contributed to adverse outcomes. Consider, for example, that therapeutic advances and enhancements have improved clinical outcomes, including an increase in survival rates but the increase in longevity has allowed some CLL patients to live long enough to experience the development of RT in the form of aggressive lymphomas [5]. What is especially compelling about the manner in which the therapies used to treat CLL/CLL-RT contribute to the frequency and type of infections that can affect this patient population. Researchers Tadmor, Welslau, and Hus (2018) point to the irony in that the benefits of therapies work to improve survival rates while at the same time allowing for the development of infections, as well as the prospect of RT [11].

The fact that the multiple available treatment regimens exist for CLL/CLL-RT that also lay the groundwork on which opportunistic infections can proliferate is daunting and presents undeniable implications for the prevention and control of these infections. Researchers suggest that at least two primary infection periods occur during CLL/CLL-RT patient therapies, the “first few months of therapy,” during which bacterial infections proliferate. This might be explained by the fact that it is the fundamental HGG that dominates as a causal factor of infections early in the presentation and treatment of CLL [11]. Treatment modalities during this period are often confined to any of the cytotoxic therapies that have been available or are currently available including but not confined to alkylator-based therapy, which contributed to bacterial infections as well as fludarabine therapy and the like [17]. The second infection period manifests a transition from single cytotoxic therapy to a mix of two or more therapies that support a “profound and sustained immunodeficiency,” sufficient to contribute to the development of opportunistic infections like “listeria, herpes simplex virus, and pneumocystis,” to name only a few [11].

On the question of how these and similar infections are prevented and controlled beyond the replacement of immunoglobulin, it is necessary to match them with the treatment regimens that fostered them in the first place and for which prophylaxis strategies have been developed. Although an examination of every infection that could be associated with the opportunistic infections that come out of the treatment for CLL/CLL-TR is beyond the scope of the present analysis, it is possible to look at those few agents identified within the two most prominent periods of CLL/CLL-RT treatment including listeria, herpes simplex virus, and pneumocystis [17]. The infections that could come from agents like the pneumocystis pneumonia, and herpes simplex viruses as well as the listeria bacterial infection that were fostered by the fludarabine therapies could be prevented and controlled with a prophylactic antimicrobial agent like the antiviral prophylaxis acyclovir as well as antibacterial prophylaxis in the form of antibiotic that could be used for preventing infection from the listeria.

Vaccines

Vaccines have historically been used to support the safety of patients with CLL/CLL-RT and vaccines for seasonal flu and pneumococcal diseases are recommended annually, however, during certain treatments, for example, vaccines should not be taken during an “anti-CD20 monoclonal antibody therapy” [13]. Understanding when and how infection prevention and control protocols work can make the difference between life and death for CLL/CLL-RT patients. This assertion is illustrated by the recommendation for early timing of seasonal flu shots for CLL/CLL-RT patients, which can benefit them by providing the best opportunity to avoid a less than optimal vaccine response [13].

As much as immunizations are perceived as viable options for preventing infection in CLL patients, including those for whom the disease has progressed to RT, their recommendation also comes with the understanding that they do not work as they work with otherwise immunocompetent individuals. In fact, CLL/CLL-RT patients or other patient populations with blood cancers typically do not realize the same benefits of immunizations as patient populations that are not already immunosuppressed [8].

Even immunizations like the coronavirus disease 2019 (COVID-19) vaccines have unique implications for CLL/CLL-RT patients. The prospects for surviving as a severe acute respiratory response to the COVID-19 are significantly less for CLL/CLL-RT patients than for otherwise healthy immunocompetent individuals, which makes understanding when and how this patient population should be immunized to protect themselves from a COVID infection critical to their survival. The issue for CLL/CLL-RT patients concerning the COVID vaccine is the same as it is with other vaccines, which is the risk of suboptimal humoral response to the vaccination [18]. And while it may be the protracted use of ever-advancing new treatments for CLL/CLL-RT patients, it is still the prolonged exposure to those treatments that contribute to the risk for this patient population to find themselves unable to “mount the appropriate response” to the COVID vaccine. Protecting CLL/CLL-RT patients from opportunistic infections at every opportunity is a challenge, not only for patients but also for the industry. Ensuring that immunocompromised patients have the safest and most effective seasonal flu vaccine, for example, was sufficient to garner debate and interest in hematology and oncology in 2014 when US pharmaceutical companies investigated with keen interest the reportedly greater safety and immunogenicity of high-dose influenza vaccines being made in Europe [18].

The impact of Richter transformation

There is little question that RT in CLL can present significant challenges to patients, despite the advances that have been made in terms of clinical outcomes for this population. Consider, for example, that while the survival rate for CLL patients has improved over the last two decades, the increased number of years translates to increasingly greater odds of developing RT for CLL patients. It is already clear that RT presents a number of challenges to CLL patients and their families. An investigation of prevention and control of infection in CLL patients with RT would be incomplete if it did not demonstrate the pursuit of information on what role RT might play in enhancing or undermining the pursuit of infection prevention and control in CLL/CLL-RT patients. This merits the identification of research establishing or discounting the need for different methods of infection prevention and control as a consequence of the progression to RT rather than the established methods already in place for CLL patients in general. A case analysis presented by researchers Baral et al. (2021) was largely the only piece of published literature to reference RT in connection to CLL and infection [5].

The case in question details the development of a “life-threatening fungal infection” in the lower leg of a CLL-RT patient, which had undergone Richter transformation, making treatment essential. As it stands, cytotoxic chemotherapies continue to reign as the most effective mechanism for treating CLL and in some cases more effectively eliminating RT Hodgkin’s lymphoma in CLL patients than the host’s brand. This is where the interest in the prevention and control of opportunistic infection with Richter transformed CCL originates, which is that it becomes the catalyst for not only the treatment of CLL and the manifestation of RT but also for the prevention and control of the infections that those treatments make CLL/CLL-RT patients vulnerable to.

Limitations

This review mainly focuses on the prevention aspect of infection in CLL patients, especially in the vulnerable population, and does not discuss other complications or clinical features of CLL. Study designs analyzed for this report include review articles, book chapters, and case reports. A universal recommendation could not be established due to a lack of observational and experimental investigations, which is why more patients at a worldwide level should be studied.

## Conclusions

Infections are a leading source of morbidity and mortality in CLL patients. Patients are prone to these infections as a result of immunological deficiencies associated with the primary disease, as well as treatment. While it is reasonable to suggest that all cancers are important when it comes to assisting those who are afflicted with them in maintaining a high quality of life, even if they are in their 70s, have one of the most common hematological malignancies in the Western Hemisphere and are so vulnerable that it is not just the many opportunistic infections that one must worry about each day, it is also the prospect that one is manufacturing a second malignancy in one’s own body. RT makes CLL not only a unique disease but also a frightening one that requires special attention to infections that can be deadly. Working along with primary physicians and experts to understand if a vaccine that continues to make headlines is as safe as they say is also a crucial part of preventing and controlling infections. In individuals with HGG or a history of infection, or both, IGRT has been shown to lower the prevalence of bacterial infections and subsequent hospitalizations. Patients with CLL should be closely examined to see whether IGRT can help them mitigate these infections. CLL is not the most consequential malignancy in the world, however, an investigation into how to prevent and control infections that exploit the vulnerabilities of those who have it makes it a very close second, and prophylactic measures are to be taken routinely even in rare infections if encountered. As clinicians, it is also vital to explore and include all differential diagnoses when dealing with rare infections in pursuit to alleviate complications with an ultimate goal to prevent mortality by employing prompt treatment options.
